# Justification for Rhinoseptoplasty in Children – Our 10 Years Overview

**DOI:** 10.3889/oamjms.2016.080

**Published:** 2016-08-01

**Authors:** Gabriela Kopacheva-Barsova, Nikola Nikolovski

**Affiliations:** *University Clinic for Ear, Nose and Throat, Faculty of Medicine, Ss Cyril and Methodius University of Skopje, Vodnjanska 17, Skopje, MK 1109, Republic of Macedonia*

**Keywords:** septo/rhinoplasty in children, indications, surgical procedures, nasal growth, midfacial development

## Abstract

**BACKGROUND:**

Nasal septal surgery and rhinoplasty are controversial in children. Traditionally, an attitude of restraint has been employed by most surgeons till an empirical age of 16 to 18 years. This is to avoid the possible adverse effects that the growth spurts may have on the nose and midface region.

**AIM:**

The aim of this paper was to present the results of rhinoplasty in children in order to restore the anatomy and function or to promote normal development and outgrowth of the nose.

**MATERIAL AND METHODS:**

Ninety seven children aged 6-14, with severe nose deformities and breathing problems through the nose, were admitted for septo/rhinoplasty at the University Clinic for Ear, Nose and Throat, Faculty of Medicine, Ss Cyril and Methodius University of Skopje, Republic of Macedonia. At our Clinic, they have been observed and photographed (with parent permission) in the period of 10 years (2006-2016). The most frequent cause of these deformities was the nasal trauma in early childhood which was ignored or untreated. All of them rhino/septoplasty were indicated in accordance with the above-mentioned recommendations for rhino/septoplasty in early childhood and in adolescents.

**RESULTS:**

In 51 children and adolescents septoplasty were prepared. Mostly there was a group of younger children age from 6-10 (68%) and adolescents (32%). In the other 31 children and adolescents, septorhinoplasty was prepared. Mostly there were children older than 12 years old and adolescents (70%). Only 30% were younger than 12 years, of course with severe nasal breathing problems, nasal septal deformities and deformities of the nasal pyramid.

**CONCLUSION:**

The growth centres of the nose have to be avoided if possible; long-term nasal issues will theoretically be minimised. If the surgeon replaces it, the cartilage of the nose becomes straighter but still intact.

## Introduction

There has been a lot of debates and controversy about the rhino/septoplasty in children and adolescents because for a long time it has been generally accepted that surgery of the bony and cartilaginous nasal pyramid and the especially nasal septum is justified until a young patient has reached the minimum age of sixteen years.

Frequently the deformities of the nasal septum and nasal pyramid occur due to the fractures of the facial and nasal bones especially in children younger than five years of age. The incidence increases with increasing age and peaks between 16 and 20 years. Numerous observations on the retarded growth of the nose after submucous resection caused the above-mentioned restriction in surgery at a young age. If the septum is determined to be the reason of nasal obstruction in a childhood, a clinical dilemma arises. Recent basic researches, assessment the results of the thesis of the negative influence of surgical trauma in growth and development of nasal and skull bone.

The behaviour of hyaline cartilage of the human nose appeared to be comparable to that of other mammals. Cartilage, although resilient, can be easily fractured whereas its tendency to integrated healing is very low, even when the perichondrium has been saved. Also surgical procedures, like in septoplasty – may result in growth disturbances of the nasal skeleton like recurrent deviations or duplicature. Loss of cartilage, as might occur after a severe septal trauma, is never completely restored despite some cartilage regeneration. Still, there remains a lack of consensus in the literature concerning the developmental effects of rhino surgery in children [1-3].

Clinical reports have not produced solid evidence for the statement that septal surgery has no negative effect on nasal growth or can serve for correcting abnormal growth. The functional and esthetic problems of the patient, however, mean a continuous stimulus for further clinical and experimental investigations [4, 5].

### Developmental aspects of the children nose

Children’s nose is different than in adults. The children nose is growing and anatomical structures are developing, therefore, there are differences in size, form and structure of supporting cartilaginous and bony framework. The anatomy of the nasal skeleton is specific and different than in adults, and the wound healing capacity of nasal cartilage is poor. The restricted wound healing capacity of septum cartilage is the essential factor in limiting the effectiveness of surgical interventions and should be recognised during the planning and surgical intervention. Accidental or surgical injury have immediate and late consequences for the further growth of the midface [6-8].

Children nose is smaller than adults, it has shorter nasal dorsum, less projection of the nasal tip and columella, rounder nostrils and a larger nasolabial angle. It has a flat tip with a shorter columella. The tissue of the nose is very soft and has a thicker subcutaneous layer. The septum of the children nose is the main is the main supporting mechanism of the nasal skeleton and it’s cartilaginous. The septum forms a T-bar-shaped structure with the upper laterals. Upper lateral cartilages extended to reach the anterior scull base. In adults, upper lateral cartilages extend cephalically underneath the nasal bones.

**Figure 1:**
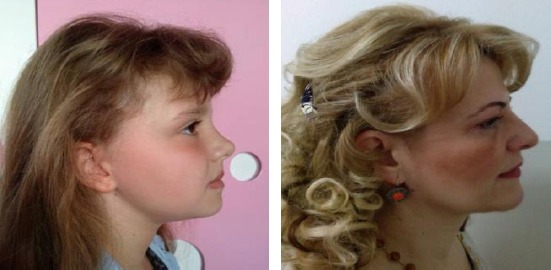
Facial profile of a child (7 years old) and her mother (44 years old). The differences in the proportions and size of the nose, facial and brain scull are obvious. The infant’s face shows smaller vertical dimensions, less frontal projection of the nose and a larger nasolabial angle (with the permission of parents)

Nasal growth is continued after puberty. Later, in the adolescents, the process ends, for male 18-20 years old, and 16-18 years old for female [9, 10] (Fig. 1).

The septo-dorsal cartilage is essential in the development of the midfacial bony and cartilaginous skeleton because growing cartilage and growing bone are in interaction. Interventions, surgical procedures, incisions on the septum cartilage, fractures, defects, interfere with normal development of the premaxilla and can seriously affect the development of all nasal supporting structures. This, special zones have special functions in the postnatal growth of the midface. The growing dorsolateral cartilage is interacting with the sutural growth of the midfacial skeleton.

There is two significant nasal growth spurts The first postnatal year which starts an endochondral ossification process (in the region of the anterior skull base) and the period of puberty when the nose grows faster compared to other periods in life.

Later, the perpendicular plate extends due to progressive ossification of nasal septal cartilage. Due to this ossification process, most of the cartilaginous part of the nasal septum loses contact with the sphenoid. The Vomer as a formation is the result of extra cartilaginous ossification.

There are two very important Growth zones responsible for the growth and development of the nose and nose elements. These two thicker areas with different mitotic activity and histological maturation are in the cartilaginous part of the nasal septum. These “growth zones”, are extended from the sphenoid [11, 12].

First, the “sphenodorsal” zone is located between the sphenoid and the nasal dorsum. This zone is primarily responsible for the normal increase in length and height of the nasal dorsum. The second, “sphenospinal” zone is located between the sphenoid and the anterior nasal spine and is the driving force in the forward outgrowth of the (pre)maxilla region.

Common causes of the destruction of zones are septal hematomas and nasal septal abscess formation, surgery or trauma. That affects the midfacial growth related to the children age and thus it can cause deformities in young children, like saddle nose deformity or/with columellar retraction, or an over-rotation of the nasal tip and a retroposition of the midface [13, 14].

### Septum surgery in children- when is justified

Rhinologic, orthodontic and cephalmometric data should be essential elements in the follow-up of children after injury and surgery of the nose. The effects of surgery (or injury) should be specified for subgroups of children with different ages, like 3–6, 7–10 years etc.

**Figure 2:**
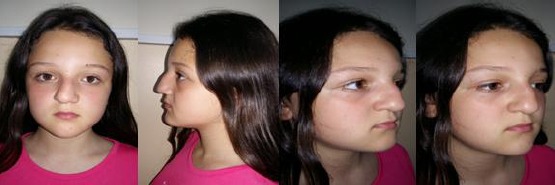
Making photographic documentation is important to define qualifications as normal or abnormal, which are in essence subjective (with the permission of parents)

Patient (child) and parents should be informed of the potential benefits of the surgery, and of continuing a follow-up of the facial growth till after the adolescent growth spurt [15, 16] (Fig. 2).

### Indications for rhinosepoplasty in children

The aim to perform rhinoplasty in children is to restore the anatomy and function or to promote normal development and outgrowth of the nose. For every indication, the expected benefits of intervention should be weighed against the possible adverse outcomes on nasal and midfacial growth. In the ideal situation, surgery should be postponed till after the pubertal growth spurt. However, there are distinct indications for immediate intervention. Apart from malignancies, these indications include the destruction of the nasal skeleton jet, it seems neither possible not even advisable to act strictly to this rule. Rhinoseptoplasty in children can be required for certain reasons.

Actual indications are severe congenital malformations of the nose, recent traumatic deformities, external distortion of the nose due to septal abscess, breathing problems due to septum pathology, cleft lip nose, dermoid cysts.

**Figure 3:**
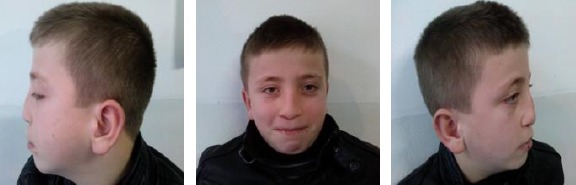
Saddle nose (rhinolordosis) due to untreated septal hematoma after the nasal trauma in early childhood) (with the permission of parents)

Children with less evident pathology have to follow-up the progression of the pathology for a certain period before making a definite decision for surgery indication. The tendency towards rhino/septoplasty in children has increased considerably. This changes in attitude bear some certain risks, so is appropriate to review once again the methods of assessment and management of pathology of the nasal skeleton in children [17, 18] (Fig. 3).

### Recommendations for Rhinosurgery in children

Before any surgery on the child’s nose is performed, it is very important first to Identify defects (older or recent) and fractures of the septum, and their relation to the specific growth and support zones.

Indications for immediate intervention are malignancies, septal abscess (septal haematoma), severe nasal trauma, cleft lip or progressive distortion of the nose. In all other cases, the surgeon has to mobilise the deviated or overlapping cartilaginous fragments, to adapt form and size of the fragments, to reconstruct a straight septum in the midline, always avoiding incisions through the growing and supporting zones (spheno-ethmoido dorsal zone).

Mobilisation of nasal bones in combination with the reconstruction of a malformed septum is the risk of postoperative instability of the (corrected) supporting structures. Intraseptal blood collection should be avoided in order to minimise the chance of infection and subsequent cartilage necrosis. In exceptional cases, it is necessary to use a 2mm osteotome to produce a satisfactory alignment of the nasal bones. Nasal packing for a few days is only tolerated by older children. Alloplastic or biomaterials are not capable of growth and implanted in a growing septum may disturb septal growth [19, 20].

The aim of this paper was to present the results of rhinoplasty in children in order to restore the anatomy and function or to promote normal development and outgrowth of the nose.

## Materials and Methods

Children aged 6-14, with severe nose deformities and breathing problems through the nose, were admitted for septo/rhinoplasty at the University Clinic for Ear, Nose and Throat, Faculty of Medicine, Ss Cyril and Methodius University of Skopje, Republic of Macedonia. At our Clinic, 97 children have been observed and photographed (with parental consent) in the period of 10 years (2006-2016). The most frequent cause of these deformities was the nasal trauma in early childhood which was ignored or untreated. All of them rhino/septoplasty were indicated in accordance with the above-mentioned recommendations for rhino/septoplasty in early childhood and in adolescents.

Fifty-eight percentages of the children were observed for the period of at least 5-7 years after the operation. The remaining patients could not be observed because they were unavailable, changed the place of living or telephone number.

## Results

At the University Clinic for Ear, Nose and Throat, Faculty of Medicine, Ss Cyril and Methodius University of Skopje, Republic of Macedonia, 97 children have been observed in the period of 10 years (2006-2016).

Children were on age 6-14 years old and were divided into 2 groups: the First group were from age 6-10 years old (36%), and second from 11-14 years old (64%). 52 of them were women and 45 were men. According to the nationality, 49 % were Albanian, 42% were Macedonian and 6% were from other nationality (Table 1).

**Table 1:** Distribution of the children patients by demographic characteristic

Demographic characteristics		
Gender	Women	52 (53.61%)

	Men	45 (46.39%)

Nationality	Macedonian	42 (43.3%)

	Albanian	49 (50.51%)

	Other nationality	6 (6.18%)

Comparing with the severity of nose deformity, nasal septal deviation and radiological investigation (CT scans or plane Radiographs founding’s) children and adolescents were divided into 5 groups. In our study, we have been made the systematic classification of the deviations of the nasal septum taking into consideration not only the classifications in the region of the cartilaginous septum but the position of the nasal septum regarding the external configuration of the nose. The objectives of this classification were to encompass all the pathological alterations of the nasal septum and to document them, in order to implement an adequate surgical technique.

**Figure 4:**
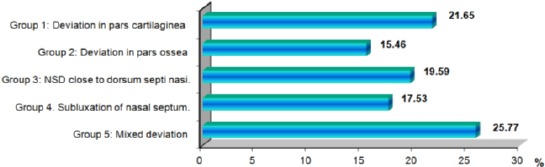
Classification of the deviations of the nasal septum in children by groups

We classified the septal deviation in 5 groups: Group 1: 21 patients were with deviation in pars cartilaginea in area of spina septi nasi (anterior parts of nasal septum); Group 2: 15 patients were with nasal septal deviation in pars ossea (posterior parts of nasal septum including vomer); Group 3: 19 patients were with nasal septal deviation close to dorsum septi nasi; Group 4. 17 patients were with subluxation of nasal septum; and Group 5: 25 patients were with mixed deviation: spina septi nasi with deviation in pars cartilaginea and nasal septal deviation in pars ossea (Fig. 4).

According to the severity of the nose deformities and breathing problems septo/rhinoplasty were indicated in all of them. But in only, 82% septo/rhinoplasty were prepared of course with parental consent and in accordance with the above mentioned recommendations for rhino/septoplasty in early childhood and in adolescents. In 51 children and adolescents septoplasty were prepared. Mostly there was a group of younger children age from 6-10 (68%) and adolescents (32%). In the other 31 children and adolescents septorhinoplasty were prepared. Mostly there were children older than 12 years old and adolescents (70%). Only 30% were younger than 12 years, of course with severe nasal breathing problems, nasal septal deformities and deformities of the nasal pyramid (Table 2).

**Table 2:** Distribution of the patients by age and type of operative technique

Distribution of the patients by age and type of operative technique		
Septoplasty N=51	6-10 years	35 (68.6%)

	11 > years	16 (31.4%)

Septorhinoplasty N=31	< 12	9 (29.03%)

	12>	22 (70.97%)

### Case Reports

**Figure 5:**
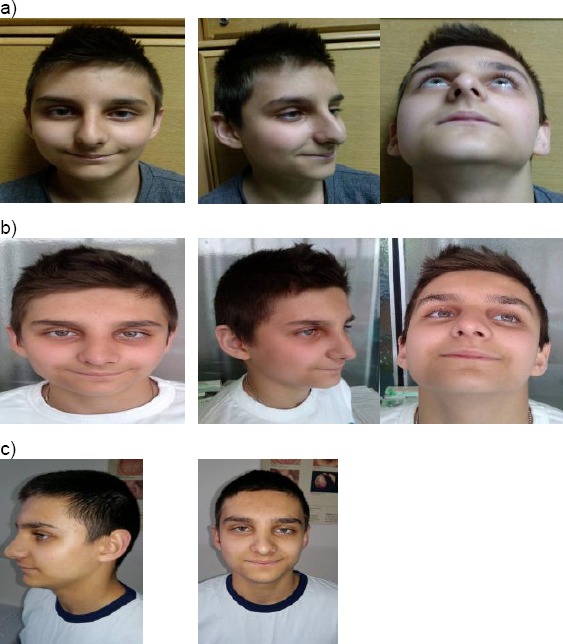
Eight years old boy with DSN. a) Pre operative results; b) 6 months after surgery; c) Two years after surgery (with the permission of parents)

Case 1: (8 years old boy) DSN. Rhinokyphosis (nasal trauma in early childhood). Nose under the tension of the long septum. Closed approach. Septal medioposition. Medial and lateral osteotomy. Cranial rotation and refinement of the nasal tip. Nasal hump reduction (Fig. 5).

**Figure 6:**
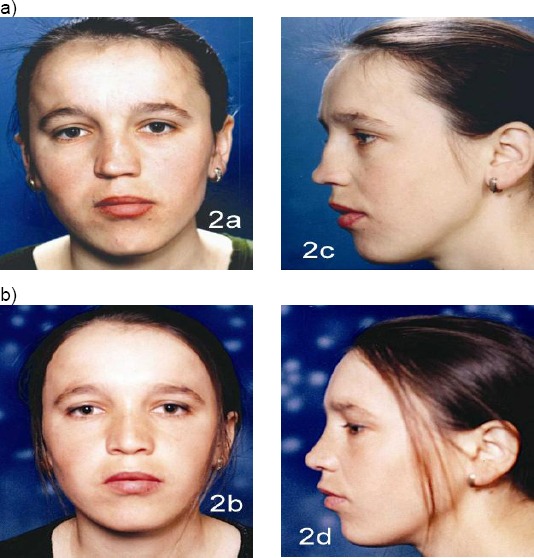
Twelve years old girl. a) Pre operative results; b) 1 year after surgery (with the permission of parents)

Case 2: (12 years old girl). Tip asymmetry. Subluxation of nasal septum. Closed approach. Septal medioposition. Cranial rotation and refinement of the nasal tip. Preservation with the septal graft in the position of the nasal dorsum at a high level (Fig. 6).

**Figure 7:**
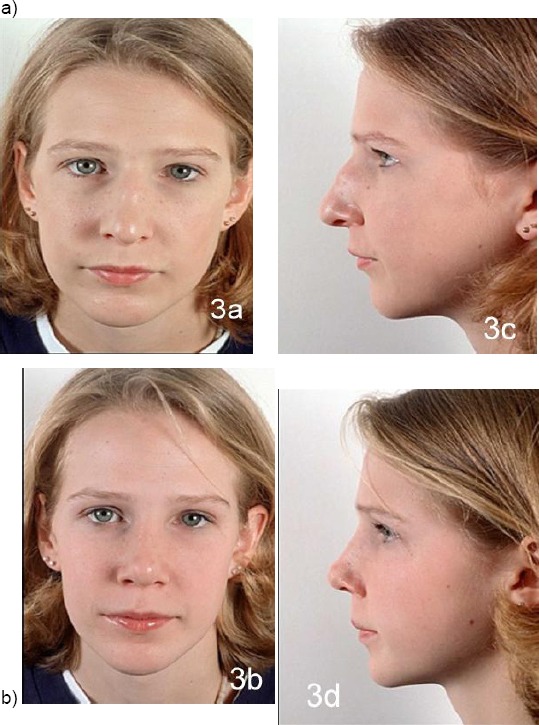
Eleven years old girl with DSN. Rhinokyphosis after nasal trauma in early childhood. a) Pre operative results; b) after surgery (with the permission of parents)

Case 3: (11 years old girl) DSN. Rhinokyphosis (11 years girl/after nasal trauma in early childhood). Nose under the tension of the long septum. Closed approach. Septalmedioposition. Medial and lateral osteotomy. Cranial rotation and refinement of the nasal tip. Nasal hump reduction (Fig. 7).

**Figure 8:**
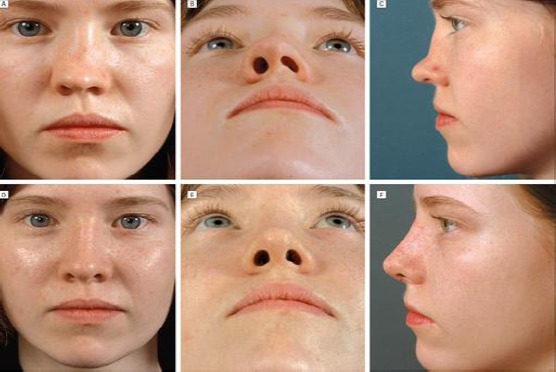
Fourteen years girl. Rhinoscoliosis and rhinolordosis (after nasal trauma). Graft (from auricula) augmentation on the nasal dorsum (with the permission of parents)

Case 4: Fourteen years girl. Rhinoscoliosis. Rhinolordosis (after nasal trauma). Closed approach. Medial and lateral osteotomy. Septal media position. Graft (from auricula) augmentation on the nasal dorsum (Fig. 8).

## Discussion

The aim to perform rhinoplasty in children is to restore the anatomy and function or to promote normal development and outgrowth of the nose. The ideal situation for every surgeon is to wait for the surgery to be performed till after the pubertal growth spurt.

A correct diagnosis of fracture or dislocation of the bony pyramid is more difficult than in adults. The examination should be repeated after 3-5 days, when the soft tissue swelling has diminished. General anesthesia is necessary. Repositioning of fractured or deviated nasal bones is nearly always possible without an open reduction. Septal hematoma and abscesses should be treated as in adults. In case when nasal cartilage is destructed - should be made a direct reconstruction using autologous cartilage grafts.

The behaviour of hyaline cartilage of the human nose appeared to be comparable to that of other mammals. Cartilage, although resilient, can be easily fractured whereas its tendency to integrated healing is very low, even when the perichondrium has been saved. Also surgical procedures, like in septoplasty – may result in growth disturbances of the nasal skeleton like recurrent deviations or duplicature. Loss of cartilage, as might occur after a severe septal trauma, is never completely restored despite some cartilage regeneration.

Often, difficult septal deformations in children are followed with deformation of nasal pyramid (rhinoscoliosis, rhinolordosis). In those cases, we cannot solve septal pathology without nasal pyramid intervention in the same time and opposite. Only severe deformities of the septum or the nasal pyramid, mostly in older children are treated by conservative septoplasty or septorhinoplasty [21-24].

Whatever type of nasal surgery should be performed in the children, the parents and the young patient should be informed, because the late results cannot be predicted. Because of the growing nose in this sensitive age, the recurrent septum pathology may occur after several years. So, information should be given about long follow-up and the possibility of a second operation after the adolescent growth spurt [25, 26].

In summary, the growth centres of the nose have to be avoided if possible; long-term nasal issues will theoretically be minimised. If the surgeon replaces it, the cartilage of the nose becomes straighter but still intact.

## References

[1] Poublon RML, Verwoerd CDA, Verwoerd-Verhoef HL (1990). Anatomy of the upper lateral cartilages in the human newborn. Rhinology.

[2] Verwoerd CDA, van Loosen J, Schutte HE, Verwoerd-Verhoef HL, van Velzen D (1989). Surgical aspects of the anatomy of the vomer in children and adults. Rhinology.

[3] Potsic WP, Cotton RT, Handler SD, Zur KB (2016). Surgical Pediatric Otolaryngology.

[4] NolstTrenite GJ, Verwoerd CDA, Verwoerd-Verhoef HL (1987). Reimplantation of autologous septal cartilage in the growing nasal septum I. The influence of resection and reimplantation of septal cartilage upon nasal growth: an experimental study in growing rabbits. Rhinology.

[5] Graber TM (1966). Postnatal development of cranial, facial and oral structures: the dynamics of facial growth. Orthodontics: Principles and Practice.

[6] Pirsig W (1979). Morphological aspects of the injured septum in children. Rhinology.

[7] Verwoerd CDA, Verwoerd-Verhoef HL (2007). Rhinosurgery in children: Basic Concepts. Facial Plastic Surgery.

[8] Pirsig W, Mladina R, passali D (2000). The influence of trauma on the growing nose. Pediatric Rhinology. Siena Tipografia Sense.

[9] Menger DJ, Tabink I, NolstTrenite GJ (2007). Treatment of septal hematomas and abscesses in children. Facial Plastic Surgery.

[10] Killian G Beitragezur sub submukosenfensterresektion der nasenscheidewand. Passow U. Schaefer Teits.

[11] Menger DJ, Tabink I, NolstTrenite GJ (2008). Nasal septal abscess in children, reconstruction with autologous cartilage grafts on Polydioxanone Plate. Arch Otolaryngol Head Neck Surg.

[12] Menger DJ, Tabink IC, Trenité GJ (2008). Nasalseptal abscess in children: reconstruction with autologous cartilage grafts on polydioxanoneplate. Arch Otolaryngol Head Neck Surg.

[13] Fattahi T, Steinberg B, Fernandes R, Mohan M, Reitter E.J (2006). Repair of nasal complex fractures and the need for secondary septo-rhinoplasty. Oral Maxillofac Surg.

[14] Bae JS, Kim ES, Jang YJ (2013). Treatment outcomes of pediatric rhinoplasty: the Asan Medical Center experience. Int J Pediatr Otorhinolaryngol.

[15] Verwoerd CD, Verwoerd-Verhoef HL (2010). Rhinosurgery in children: developmental and surgical aspects of the growing nose]. Laryngorhinootologie.

[16] Pediatric rhinoplasty in an academic setting (2007). Facial Plast Surg.

[17] Riechelmann H, Rettinger G (2004). Three-step reconstruction of complex saddle nose deformities. Arch Otolaryngol Head Neck Surg.

[18] Dennis SC, den Herder C, Shandilya M, NolstTrenité GJ (2007). Open rhinoplasty in children. Facial Plast Surg.

[19] Septoplasty pearls (2009). Dobratz EJ, Park SS. Otolaryngol Clin North Am.

[20] Kühnel TS, Reichert TE (2015). Trauma of the midface. GMS Curr Top Otorhinolaryngol Head Neck Surg.

[21] El-Hakim H, Crysdale WS, Abdollel M, Farkas LG (2001). A study of anthropometric measures before and after external septoplasty in children: a preliminary study. Arch Otolaryngol Head Neck Surg.

[22] Dennis SCR, den Herder C, Shandilya M, NolstTrenite GJ (2007). Open rhinoplasty in children. Facial Plastic Surgery.

[23] Bradbury E (2012). Meeting the psychological needs of patients with facial disfigurement. Br J Oral Maxillofac Surg.

[24] Boenisch M, Hajas T, NolstTrenite GJ (2003). Influence of Polydioxanone foil on growing septal cartilage after surgery in an animal model. Arch Facial Plast Surg.

[25] Verwoerd CDA, Verwoerd-Verhoef HL, Sih T, Clement PAR (2005). Anatomy and development of the nasal septum in relation to septal surgery in children. Pediatric Nasal and sinus disorders.

[26] NolstTrenite GJ, NolstTrenite GJ (2005). Postoperative care and complications. Rhinoplasty.

